# Change and stability: Within-person life satisfaction over a 20-year period using data from the HUNT survey

**DOI:** 10.1177/1403494820957439

**Published:** 2020-09-20

**Authors:** Frode Lysberg, Tomas Bjerregaard Bertelsen, Cathrine Lysberg, Magnhild Høie, Geir Arild Espnes, Siw Tone Innstrand

**Affiliations:** 1 NTNU Center for Health Promotion Research, Norwegian University of Science and Technology, Norway; 2 Department of Psychosocial Health, Faculty of Health and Sport, University of Agder, Norway; 3 Regional Hospital of Southern Norway, Norway; 4 Faculty of Medicine, Pomeranian Medical University, Poland; 5 Department of Psychology, Norwegian University of Science and Technology, Norway

**Keywords:** Within-person life satisfaction, life satisfaction dynamics, set point, well-being, cohorts, age groups

## Abstract

*Background:* The aim of the present study was to investigate within-person life satisfaction (LS) dynamics for two age groups, 20–29 and 30–39 years, from 1984 to 1986 and to follow them over a 20-year period. *Methods:* Data from 1984 to 2008 were extracted from the large, prospective, longitudinal North-Trøndelag Health Study (HUNT), Norway. This paper includes data from more than 14,500 persons. Data were analysed using logistic regression, and LS dynamics were modelled using gender, time and self-rated health. *Results:* The analyses revealed that about 20% of people in these age groups had a stable level in LS, also known as set point. Long-term LS change, defined as ⩾2 SDs, was reported for 9% and 6% of people in the youngest and oldest age groups, respectively. A large proportion of more than 70% of people had fluctuations in their LS over a 20-year period. A significant decrease in within-person LS was seen for the age groups from 1984–86 to 1995–97 where a significant increase appeared from 1995–97 to 2006–08. For the initial 20–29 age group, the odds of having a higher score increased by 34%, and for the initial 30–39 age group, the within-person LS increase was 81%. Self-rated health was the most crucial variable influencing within-person LS. ***Conclusions:* These findings suggest that a significant proportion of the responders had a long-term within-person LS change over the 20-year period.**

## Introduction

Questions about life satisfaction (LS) dynamics have been heavily debated over recent decades [1, 2], especially the question of how LS can vary around an individual’s set point [1], what factors contribute to change, and to what extent LS can change [3]. Data indicate that LS can be of great importance for individuals and societies as a whole, and several studies suggest that a high level of LS is associated with a better quality social life, greater occupational success, better relationships, improved health, delayed mortality and both higher productivity and income [3-6]. A large body of data also indicates that happy individuals promote happy societies and economically productive and healthy nations [6, 7]. Several studies indicate that LS has increased in the last decades for populations in several Western countries [1, 6]. A paper looking at LS dynamics for age cohorts from 20 to 70+ years in Norway found an increase in LS of about 16% for the odds of having a higher score for younger age groups (20–39 years), and about 32% increase in the odds of having a higher score for middle and older age groups (40–69 and 70+ years) over 20 years [8]. Veenhoven and Hagerty [9] state that economic growth increases happiness, and they showed an increase in economic growth and happiness in 14 of 21 nations studied. However, when looking at a change in within-person LS, set-point theory holds that adult LS is very stable over time, even if people are faced with challenging life events or substantially changed circumstances. This theory states that people will return to their baseline – their set point – a phenomenon also referred to as ‘the hedonic treadmill’ [2, 10]. Why this stability in LS occurs has been linked by several authors, among other things, to stable personality traits, especially neuroticism and extroversion [1, 11]. LS is in this paper is defined as the broad cognitive aspect of the higher order of well-being [12, 13].

Long-term panel data are stated to be the best way to follow individual LS trajectories, and results from long-term studies have strongly challenged set-point theory [1, 3, 14]. Results from these studies indicate that a large number of respondents in Western countries have substantial and long-term changes in LS [15-17]. One paper looking at long-term change in within-person LS found that 24% of the responders changed significantly in over 15 years [16]. Based on new LS dynamic results, many researchers today accept that set-point theory is inadequate [1, 3, 14]. Moreover, in 2018, Headey and Muffels [1] published a paper introducing a new LS theory for Western societies: the paper outlines an LS trajectory of stability, change and volatility. In their paper, they state that long-term stability of LS is similar to the set-point theory proposition, and 61% of their responders were characterized as having stable LS; however, 24% fell into the category of changed LS, and the rest of the responders into the volatility category, including people with inter-individual differences in their LS [1]. It is now essential to fill the gap for the new theory postulations about LS dynamics. This paper explores the within-person LS trajectories of two age groups 20–29 and 30–39 years from baseline in 1984–86 for more than 14,500 people over 20 years, and also the factors influencing within-person LS dynamics. We hypothesized that for both age groups, a significant number of participants would experience a change in their within-person LS over the 20-year period.

## Methods

### Sample

The study samples were obtained from the health survey of North Trøndelag (HUNT) [18]. It consists of prospective, population-based longitudinal studies that were conducted from 1984 to 2008. These surveys were conducted in three study sets: 1) HUNT 1 (1984–1986; including 77,212 participants; 2) HUNT 2 (1995 –1997; including 65,237 participants); and 3) HUNT 3 (2006–2008; including 50,807 participants). More details of the HUNT studies are described elsewhere [18-20]. In the present study, a subsample of HUNT was selected containing those individuals who were between ages 20–39 in HUNT 1 and had answered the LS question also for HUNT 2 and HUNT 3. A total of 14,531 persons were included. For the age group 20-29 years, 5,561 responders participated through all HUNT study sets, counting for 38.3% of the participants, and for the age group 30–39 years, 8,970 responders participated in all HUNT study sets, counting for 61.7% of the participants. This paper used an analysis of within-subject change to reduce respondents lost due to attrition. The data in Table I report the number of responders LS, and the data in Table II report the responder’s stability, volatility, positive/negative LS change. The HUNT studies were approved by the Regional Committee for Medical and Health Research Ethics of Norway and approved by the Norwegian Data Protection Authority.

**Table I.** Number of responders at the individual level for different levels of LS and gender.

HUNT	HUNT 1				HUNT 2				HUNT 3			
Gender	Male		Female		Male		Female		Male		Female	
	*n*	%	*n*	%	*n*	%	*n*	%	*n*	%	*n*	%
Very satisfied	1264	19.2	1701	21.4	731	11.1	995	12.5	1197	18.2	1445	18.2
Satisfied	2032	30.8	2630	33.1	2063	31.3	2667	33.6	2456	37.3	2920	36.8
Somewhat satisfied	2514	38.1	2765	34.8	2760	41.9	3021	38.1	2101	31.9	2538	40.0
Dissatisfied	783	11.9	842	10.7	1039	15.7	1255	15.8	839	12.6	1035	5.0
Total	6593	100	7938	100	6593	100	7938	100	6593	100	7938	100

**Table II.** LS dynamics for within-person LS from 1984 to 2008.

LS dynamics over 20 years 1984–2008	Age group 20–29 in 1984–1986 (%)	Age group 30–39 in 1984–1986 (%)
LS – Stability/set point	18	22
LS – Volatility	73	72
LS – Positive change	4	3
LS – Negative change	5	3

LS dynamics for the two age groups. Change was estimated as ⩾2 SD. All estimates were statistically significant (*p* < 0.01).

### Measures

LS was measured using a single-item question, administered in all HUNT sets, which asked the respondents to indicate how satisfied they were with their life as a whole. This question was reviewed and is considered to have adequate reliability and validity [19, 21]. The question was, ‘Thinking about your life at the moment, would you say that you by and large are satisfied with life, or are you mostly dissatisfied?’ The respondents used a seven-point rating scale ranging from one (very satisfied) to seven (very dissatisfied). In our analysis, we reduced the seven-point scale to four points, to ensure convergence of the model. More specifically, the neither satisfied nor dissatisfied, somewhat dissatisfied, dissatisfied, and very dissatisfied responses were combined into one category and labelled ‘dissatisfied’ since few responses indicated such high degrees of dissatisfaction. In our analysis, we reversed the direction of the scale so it ranged from one (dissatisfied) to four (very satisfied). The data in Table I report the number of responders over the three HUNT study sets. The question, ‘How is your health at the moment?’ was also reviewed and considered to have reasonable reliability and validity [20]. Data for this variable are presented in Table III.

**Table III.** LS dynamics for two age groups for all HUNT study sets.

Fixed effects	Age 20–29 in 1984–1986		Age 30–39 in 1984–1986	
	OR	95% CI	OR	95% CI
HUNT 1 (reference)	1	–	1	–
HUNT 2	0.66	0.61–0.71	0.77	0.73–0.82
HUNT 3	1.34	1.23–1.45	1.81	1.70–1.93
Gender (women as ref)	0.83	0.76–0.90	0.83	0.77–0.89
Self-rated health	5.08	4.75–5.43	5.54	5.23–5.87
Random effects of individual	Variance (log-odds scale)	Standard deviation (log-odds scale)	Variance (log-odds scale)	Standard deviation (log-odds scale)
Intercept/threshold	1.31	1.14	1.73	1.31
Slope for time	0.17	0.41	0.21	0.45

For all age groups, HUNT 1 was used as the reference group. All estimates are statistically significant (*p* < 0.01).

### Statistical analyses

In this study, within-people LS change was modelled using gender, time, and self-rated health as predictors. Associations were modelled using multilevel ordinal logistic regression in the STATA program, 2019 [22], due to a nested data-structure with within-participant repeated measures. The multilevel model was used to describe the within-person change, fixed effects, and differences between individuals in how they changed, and to describe random effects. Missing data were explored using visual inspection and the Hawkins *F*-test for non-normal missing data [23]. Our analyses are considered exploratory, so we did not adjust for multiple testing. *P*-values <0.01 were considered statistically significant. The data were disaggregated according to the procedures outlined by Wang and Maxwell [24]. The predictor variable SRH was grand-mean centred, and the gender variable was centred on being female. The results are expressed as odds ratios (ORs) with 95% confidence intervals (CIs), except for the random effects of between-person variance. This is reported as the variance in the regression coefficient on the log-odds scale with a standard deviation (SD) of this point estimate. We modelled the odds of achieving a higher level of LS using HUNT 1 as the reference. The fit of ordinal regression models was assessed using the test of parallel lines before performing the multilevel variant, and the multilevel model was assessed first with an empty model and then by gradually adding variables. The final model included fixed effects for gender, time and SRH and random effects for the intercept and for the slope of time.

## Results

In the youngest age group, 20–29 years (in 1984–86), the intraclass correlation coefficient (ICC) for the intercept was 0.59, indicating that 59% of the total variability in the threshold for change was due to between-person differences. In the age group 30–39 years (in 1984–86), the ICC was 0.64, indicating that 64% of the total variability in the threshold for change was due to between-person differences. The multilevel regression of individual change shows that both age groups had the lowest odds of increasing in LS at HUNT 2 and highest odds of increasing in LS at HUNT 3, with the 30–39 age group showing higher ORs at both time points. Furthermore, being a male decreased the OR of LS by 17% in both age groups, using women as reference. SRH was a crucial factor for within-person LS dynamics in both age groups. The between-person differences, random effects, are described in Table III.

**Figure 1.**
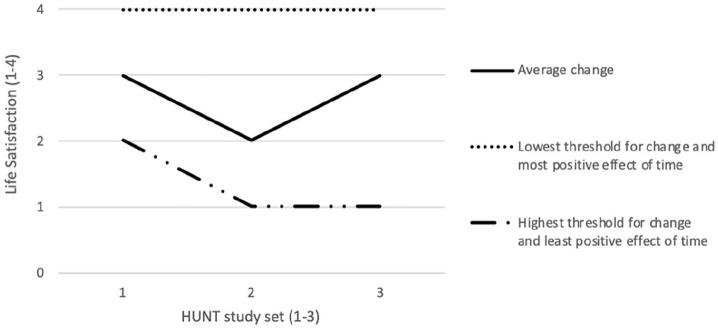
Predicted values of life satisfaction based on regression. Lowest and least refer to individuals 2 SD below the average threshold for change and effect of time respectively. Highest and most refer to individuals 2 SD above the average threshold for change and effect of time, respectively. All estimates were statistically significant (*p* < 0.01).

The random effects intercept describes the inter-individual differences of the threshold for change and effect of time of a female with average health. The variance between individuals in thresholds for change suggested that there were considerable differences between the ability to change, with some people easily gaining positive change, whereas for others it appeared almost impossible. This was similar for both age groups. Likewise, the random effects of time suggested that the influence of time was also highly dependent on the individual, with some individuals gaining positive change over time, whereas others did not. Figure 1 depicts the results of solving the regression equation for a female of average SRH from the 20–29 age group. One line depicts the average intra-individual predicted change across the three study sets, the fixed-effects portion of the regression. The two other lines depict how inter-individual differences influence change, the random-effects portion of the regression. As can be seen, the average expected change for the individual was moderate, but there was a substantial difference between individuals in how much change was predicted and in what direction.

## Discussion

This study investigated the within-person LS trajectories for a period of more than 20 years, and the results indicated that LS dynamics can be divided into three categories: long-term stability, volatility, and long-term change, as suggested by Headey and Muffels [1]. For the long-term stability category (set point in the paper by Headey and Muffels [1]), 18% of people in the initial 20–29 age group, and 22% in the initial 30–39 age group had long-term LS stability when using ⩾ 2 SD to estimate LS change. Headey and Muffels reported that approximately 61% of their participants were classified as having long-term stability [1]. However, they used grand means to estimate LS change. In research by Fujita and Diener [16], the LS of 76% of their participants did not change significantly. This is more in line with the results from the current study, where around 80% of responders either had a long-term stability LS or were in the volatility group. The percentage of people in the volatility group presented in this paper was high: more than 70% in both age groups. The volatility group did not have a significant within-person LS net change, evaluated from the starting point in 1984–86 and at the study end in 2006–08. For the age groups’ positive or negative LS change (⩾2 SD), data indicated that 8.5% of those in the 20–29 age group, and 6.4% of those in the 30–39 age group had within-person LS long-term change over 20 years. This is more in line with the results presented in the paper by Headey [25] in which he states that 14% of the persons in the study reported change in LS when using ±2 points to define change. Headey also states that changes of this magnitude are not compatible with set-point theory [25]. The data on stability, volatility, and positive/negative change are shown in Table II. As seen in Figure 1, the fitted regression predicts that some individuals, labelled ‘Lowest threshold for change and most positive effect of time’, will start with a high LS and remain that way throughout the 20-year period. On the other hand, regression predicts that some people will start out lower on LS and decrease over the 20-year period, these individuals are labelled ‘Highest threshold for change and least positive effect of time’. This paper is unable to identify the variables underlying the threshold for change in LS and the decrease in LS, due to the limited number of variables used. However, as described by Headey [25] some of the discussion regarding LS change is linked to personality traits such as neuroticism and extroversion, and major life events like marriage, first child being born, separation, divorce, a partner dying, becoming unemployed, annual household disposable income, and health problems [1, 16, 25, 26]. The current results indicated that both age groups (20–29 and 30–39 years) had a significant decrease in within-people LS of about 34% and 23%, respectively, in the odds of scoring higher for the period 1984–86 to 1995–97. However, at the end of the 1980s and the beginning of the 1990s, there was pessimism in Norway due to high unemployment, low productivity and a high inflation rate. Following a decrease in LS, data from this paper indicated that the initial 20–29 age group had an increase of 34% in the odds of scoring higher on LS from 1995–97 to 2006–08, at a time when they were about 40–49 years of age. The initial 30–39 age group had an increase of 81% in the odds of scoring higher on LS from 1995–97 to 2006–08. At that time, they were about 50–59 years of age. However, a study from Norway found that several factors such as gross domestic product (GDP), health, income, welfare, the labour market, and so forth, are important factors for LS dynamics, and from the beginning of the 1990s, most of these factors have seen positive development in Norway [27]. When LS increased for the age groups in this study (1995–97 to 2006–08), the participants had reached the age of about 40 to 60 years. These people had therefore reached an age level where studies indicate that LS can start to exhibit a U-shaped increase in LS [28, 29]. Another factor highlighted for Norway in a study by Lysberg et al. [8], is the increase in LS for all age groups from 1984 to 2008, but especially for middle and older age groups (40–69 and 70+ years). Data presented in this paper also indicated that SRH was the most important factor for within-people LS dynamics, with an increase of 408% in the odds of scoring higher on LS for the initial 20–29 age group, and an increase of 454% in the odds of scoring higher on LS for the initial 30–39 age group. The very large SRH impact on within-people LS dynamics over such a long period is interesting. The SRH question was evaluated to include the respondents’ physical, social and emotional evaluation [6]. Moreover, data from several studies indicate that health has a large impact on LS [1, 7, 30]. The results from the current study also indicated that gender had some impact on LS and that men score lower on LS using women as reference. This finding is in line with other studies [6-8]. The results presented in this paper supported the hypothesis that a significant proportion of people will have changed their within-person LS over a 20-year period.

The strength of the present study was inclusion of longitudinal data following more than 14,500 responders for more than 20 years. Furthermore, that the study followed within-person LS dynamics from young adulthood to pre-retirement. Data used in this paper are from the health survey HUNT, which has a good quality in the data collection [18]. The findings presented in this paper should encourage practitioners and public health policymakers to focus on optimizing the population’s health, particularly younger people. The study by Lysberg et al., looking at the change in SRH found a 46% decrease in the odds of scoring higher for the 20–29 age group from 1984 to 2008 [20]. This paper presents data indicating that within-person LS for a large number of participants was characterized by change and volatility over time. These results should be further investigated in future studies for a better understanding of LS dynamics and developing a new LS theory. Nevertheless, the findings presented in this paper need to be carefully interpreted. Also, some limitations must be kept in mind. First, the study was based on data from Norway, a country that ranks very high on most surveys of LS, and this may preclude our findings from being generalized to other countries [8]. Second, this study used a single variable for detecting within-person LS. Although single-item variables for LS have proven to be valid and reliable, future studies should strive for more objective well-being measures. Third, this study included a small number of variables, and our findings could have been strengthened had we included additional variables such as GDP, income, welfare, unemployment, and so on. To conclude, the results presented in this paper indicated a large and significant within-person LS change and volatility, and that health is an important factor influencing LS dynamics.

## Conclusions

Maximizing LS and health are essential for people’s lives and for promoting happy societies and economically productive and healthy nations. This study indicated that a significant number of participants have a long-term change in LS, and that LS volatility is prevalent for a large number of people. This study could play an important role in providing further data for a new theory of LS dynamics.
